# Risk of perpetrating intimate partner violence amongst men exposed to torture in conflict-affected Timor-Leste

**DOI:** 10.1017/gmh.2018.16

**Published:** 2018-07-03

**Authors:** Susan Rees, Mohammed Mohsin, Alvin Kuowei Tay, Zachary Steel, Natalino Tam, Zelia da Costa, Cesarina Soares, Wietse Tol, Valsamma Eapen, Mark Dadds, Derrick Silove

**Affiliations:** 1 Psychiatry Research and Teaching Unit, School of Psychiatry, University of New South Wales, Sydney, New South Wales, Australia; 2 St John of God Hospital, School of Psychiatry, University of New South Wales, Sydney, New South Wales, Australia; 3 Mental Health, Johns Hopkins University Bloomberg School of Public Health, 624 N Broadway, HH863, Baltimore, Maryland, USA; 4 Peter C. Alderman Foundation, Mawanda Road, Plot 855, PO Box 20129 Nakawa, Kampala, Uganda; 5 Child and Adolescent Mental Health, Liverpool Hospital, School of Psychiatry, University of New South Wales, Sydney, New South Wales, Australia; 6 School of Psychology, University of Sydney, Sydney, New South Wales, Australia

**Keywords:** Trauma, intimate partner violence, mental disturbance, torture

## Abstract

**Background.:**

A key issue in need of empirical exploration in the post-conflict and refugee mental health field is whether exposure to torture plays a role in generating risk of intimate partner violence (IPV), and whether this pathway is mediated by the mental health effects of torture-related trauma. In examining this question, it is important to assess the impact of socio-economic hardship which may be greater amongst survivors of torture in low-income countries.

**Methods.:**

The study data were obtained from a cohort of 870 women (recruited from antenatal clinics) and their male partners in Dili district, Timor-Leste. We conducted bivariate and path analysis to test for associations of men's age, socioeconomic status, torture exposure, and mental disturbance, with IPV (the latter reported by women).

**Results.:**

The path analysis indicated positive paths from a younger age, torture exposure, and lower socio-economic status amongst men leading to mental disturbance. Mental disturbance, in turn, led to IPV. In addition, younger age, lower socio-economic status, torture exposure, and mental disturbance were directly associated with IPV.

**Conclusions.:**

Our data provide the first systematic evidence of an association between torture and IPV in a low-income, post-conflict country, confirming that low socio-economic status, partly related to being a torture survivor, adds to the risk. The high prevalence of IPV in this context suggests that other structural factors, such as persisting patriarchal attitudes, contribute to the risk of IPV. Early detection and prevention programs may assist in reducing the risk of IPV in families in which men have experienced torture.

## Introduction

Torture and intimate partner violence (IPV) are pervasive forms of human rights abuses that are known to cause high rates of mental disorder amongst survivors worldwide (Steel *et al.*, 2009; García-Moreno, 2013). Although differing in key aspects, torture and IPV share some core characteristics including the intent of the perpetrator to subjugate, degrade, and disempower the victim. Consequently, survivors are commonly left with persisting feelings of humiliation, self-doubt, and emotional isolation, reactions that are interrelated with society's tendency to condone, conceal, or minimize these violations (Herman, 2015). Together, these factors may undermine the capacity of survivors to manage frustrations, leading to use of aggression in the family. Importantly, clinicians working with refugees and other conflict-affected populations have noted that IPV may be a risk in families of torture survivors, yet there are no systematic data corroborating that observation (Rees & Pease, 2006; Gupta *et al.*, 2009).

Torture remains a ubiquitous instrument of repression despite the ratification of the United Nations Convention Against Torture (Amnesty International, 2014) by the majority of countries worldwide. The ‘war on terror’ dispelled the notion that torture was a practice confined to countries ruled by oppressive regimes or in states of major conflict or chaos (McCoy, 2007). There is now conclusive evidence that torture has a major impact on the mental health and psychosocial functioning of survivors (Steel *et al.*, 2009). A systematic review and meta-regression of studies amongst refugee and other populations exposed to persecution confirmed that torture is the most potent of all the traumas of mass conflict in generating post-traumatic stress disorder (PTSD) (Steel *et al.*, 2009). In addition, PTSD in torture survivors is more likely to be severe and chronic, and is frequently complicated by symptoms of anxiety, depression, and substance misuse. For all these reasons, there is a high risk that survivors will develop major interpersonal difficulties when they return to their families.

Men survivors of torture may be at risk of enacting violence within the family for several inter-related reasons. As indicated, PTSD and related symptoms of anxiety and depression tend to be more common, severe, and chronic amongst torture survivors. Associated excessive use of alcohol and other substances further reduces frustration tolerance, and thereby increases the risk of aggression towards intimate partners (Taft *et al.*, 2017). More broadly, torture represents an assault on the person's sense of integrity and dignity, undermining the survivor's self-esteem (Basoglu, 1992), and, in the case of militants or political activists, a strongly developed masculine, warrior identity. For all these reasons, men survivors of torture may overcompensate for their sense of loss of control by re-asserting their male power within the family, which in the extreme situation, may increase the risk of IPV (Turner & Gorst-Unsworth, 1990).

A cycle of violence model relevant to post-conflict countries has been identified (Rees & Pease, 2006; Catani *et al.*, 2008; Catani, 2010; Saile *et al.*, 2013). The core hypothesis is that men exposed to the traumas of mass conflict are at risk of enacting violence against their families upon return, resulting in a cascade of adverse psychosocial effects on other members, including IPV and harsh parenting behaviours (Catani *et al.*, 2008; Catani, 2010; Saile *et al.*, 2013; Rees *et al.,* 2015). The risk is that conflict and discord in the home will increase the risk of future aggression and violence in the offspring. A key issue, however, is whether exposure to torture, a major instrument of oppression in persecutory societies, is specific in increasing the risk of violence when men survivors return to their families, and if so, whether this cycle of violence is mediated by the mental health effects of past abuse (Rees & Pease, 2011).

IPV refers to acts of physical, sexual, financial, and psychological violence that occur between people who have, or have had, an intimate relationship in a domestic setting (Devries *et al.*, 2013). There is overwhelming evidence that IPV has profound effects on the mental and physical health of survivors, the large majority being women (Coker *et al.*, 2002; Rees *et al.*, 2011; Beydoun *et al.*, 2012; Trevillion *et al.*, 2012). Although IPV is a worldwide problem, rates vary substantially across different countries and settings (García-Moreno, 2013). There is a consensus that the persistence of patriarchal values that promote and condone violence against women account substantially for this variation in the rates of IPV across countries (Heise, 1998; Jewkes, 2002; Dunkle *et al.*, 2004; Yodanis, 2004; Fulu *et al.*, 2013; Vichealth, 2014). However, not all men are perpetrators. The prevalence of non-perpetrator men in patriarchal societies suggests that other social and individual factors may be implicated (Heise, 1998). Exposure to trauma appears to be an important contributor to IPV (Heise, 1998; Rees & Pease, 2006; Stark, 2011), an inference drawn from diverse studies (Heise, 1998; Krug *et al.*, 2002; World Health Organization, 2012) focusing on the effects of childhood abuse, other forms of civilian violence, community conflict (Gupta *et al.*, 2009), and combat (Jordan *et al.*, 1992; Byrne & Riggs, 1996; Dansby & Marinelli, 1999; Orcutt *et al.*, 2003; Marshall *et al.*, 2005; Taft *et al.*, 2007; Stappenbeck *et al.*, 2014). Few studies have addressed this issue across cultures, an exception being an inquiry amongst a convenience sample of immigrant men in the USA which indicated that men exposed to political violence are at an increased risk of perpetrating IPV (Gupta *et al.*, 2009).

It is well-established that poverty is associated with risk of IPV, potentially contributing to high rates of this form of abuse in low- and middle-income countries such as Timor-Leste (Jewkes, 2002). Economic recovery may be slower for torture survivors and their families, as a consequence of discrimination experienced during the period of conflict, leading to financial hardship (Jewkes, 2002; Rees *et al.*, 2016). In Timor-Leste, political activists (who were at high risk of torture) experienced the wilful destruction of their property and possessions, exclusion from education and work opportunities, and in some cases, social ostracism (Stanley, 2008; Scambary, 2009). We therefore include a composite index of socio-economic deprivation in our analysis.

The history of conflict in Timor-Leste made it possible to examine associations of torture, socio-economic deprivation, mental disturbance, and IPV. During the Indonesian occupation (1975–99) and subsequent humanitarian emergency (1999–2000), the population was exposed to extensive human rights violations including torture, murder, arbitrary incarceration, and mass displacement (Dunn, 2009). The protracted struggle for independence culminated in a nation-wide humanitarian emergency leading up to and after a referendum on independence in 1999. Indonesian retribution after the vote led to the destruction of most of the territory's built infrastructure, the mass internal displacement of populations, and extensive human rights violations. Abuses conformed to the wider definition of torture including sustained beatings of Timorese activists and members of opposition parties by the military and militia (Dunn, 2009; Jørgensen, 2017). The Indonesian military withdrew in 1999 when the United Nations assumed control of the territory, leading up to national independence in 2002. Further periods of internal conflict ensued, culminating in a major upheaval in 2006–07 which led to factional violence, further human rights abuses, destruction of property, and the mass internal displacement of populations. The most affected region was the Dili District, the population catchment area of the present study.

Despite the introduction of domestic violence legislation and campaigns to raise awareness of the problem, there is a high level of tolerance for violence against women in Timor-Leste (Wigglesworth *et al*., 2015). Consistent with those beliefs, IPV rates are high, with estimates indicating that 34% of women experience physical abuse by an intimate partner after the age of 15 years (DHS Timor 2009–2010). In our baseline assessment of the present cohort of 1672 pregnant women we found that in the past year 30.6% (*n* = 511) experienced severe psychological abuse and one-fifth (*n* = 327) experienced severe psychological and physical abuse (Rees *et al.*, 2016).

Most of the men in our study were adolescents or young adults during past periods of conflict in Timor-Leste, a group that faced the highest risk of being targeted, apprehended, and tortured. There is conflicting evidence regarding the propensity to perpetrate IPV in younger rather than older men (Santana *et al.*, 2006; Fleming *et al.*, 2015). Overall younger age men appear to be more likely to perpetrate current violence, the prevalence reducing with age (Peters *et al.*, 2002). There is insufficient evidence related to men's age and IPV in conflict-affected populations, including whether early age exposure to torture increases the risk of future violence against female partners.

To our knowledge, the present study is the first to focus specifically on torture as a possible determinant of IPV in a culturally distinct, post-conflict country. The present study is based on the second wave of data collection amongst women and their male intimate partners. The baseline recruitment was undertaken at antenatal clinics as described hereunder. The objective was to examine the relationship between torture, socio-economic status, mental disturbance, and IPV in a low-income, post-conflict country. We tested the following hypotheses: (1) that men exposed to torture are at particular risk of enacting IPV in the post-conflict period; (2) that torture exerts its effects via symptoms of mental disturbance (related to PTSD, depression, and misuse of alcohol); (3) that socio-economic deprivation makes an additional contribution (both via mental disturbance and directly) to IPV.

## Methods

### Female participants

The data are drawn from the second wave of a cohort study of women, first recruited during the second trimester of pregnancy from the four largest public antenatal clinics in Dili district, Timor-Leste. Details of the methodology have been published elsewhere (Rees *et al.*, 2016). The clinics provide services to both urban and rural areas. At the time of recruitment, just over a fifth of the national population (1.1 million) lived in the Dili district (which includes the capital); 96% of pregnant women attended the antenatal clinics where recruitment took place (National Statistics Directorate (NSD) [Timor-Leste], 2010). We implemented a rolling recruitment strategy, our field team spending 3–4 month periods in succession at each of the four large government antenatal clinics (Becora, Bairo Pite/Vera Cruz, Comoro and Formosa) in the district.

The baseline study was conducted between June 2013 and August 2015. Excluded from the study were women with overt psychosis, profound intellectual impairment or severe medical illness requiring referral to hospital. A total of 1672 pregnant women were interviewed at baseline, a response rate of 96%.

The second wave survey of the cohort of women was conducted between March 2014 and November 2016. In the follow-up survey, 1380 women (82.5%) of the original cohort of 1672 were identified for interview and 1303 were successfully interviewed, a response rate of 94.5% of those re-contacted. The 369 non-participants at follow-up comprised women who could not be contacted despite five visits to the original dwelling and efforts to trace those who had moved (49% of non-responders); refusal (22%); relocation and could not be traced (25%); and other factors (5%).

### Male participants

At follow-up, women's conjugal partners (defined as live-in intimate male partners, noting that a strong taboo persists regarding same-sex relationships in Timor-Leste) were approached and where possible, interviewed. A total of 889 male partners were interviewed at follow-up, representing a response rate of 68.2% of those eligible (889 out of 1303).

The main reason for men's non-participation was inability to make contact despite five visits to the dwelling (*n* = 255; 19.6%). The remaining men were not included either because of miscarriage or the baby died (*n* = 77; 5.9%), or the male participant was overseas (*n* = 46; 3.5%) or he had divorced or his partner died (*n* = 36; 2.8%). In total 19 records were excluded due to extensive missing data. The final analytic sample, therefore, comprised 870 matched couples with complete data for both women and their male partners.

### Survey measures

We selected the most widely used and tested measures in the field to assess key indices of IPV and a composite index of mental disturbance. International guidelines were applied for the cultural and linguistic adaptation, translation, and back-translation of measures (van Ommeren *et al.*, 1999). Most of the measures had been applied in previous studies in Timor-Leste (Silove *et al.*, 2014). Of the full compendium of measures, we report only those used in the present analysis.

### Socio-demographic characteristics of men and women

Socio-demographic variables were adopted from the Timor-Leste census, which included: place of usual residence (urban/rural), age, sex, marital status, level of education, and employment status.

### Socio-economic index for men

The variable representing socio-economic status was based on a composite of three indices: level of education (high 1; low 0), the cut-off being whether the person had advanced to Technical College/Diploma/University education (1) or high school/lower (0); employment based on currently employed (1) or not (0); and self-reported ongoing poverty/deprivation on a multi-item index (no = 1, yes = 0). We developed and validated an index of ongoing adversity (poverty/deprivation) relevant to contemporary life in Timor-Leste based on an iterative process of qualitative interviews, field testing, and feedback by a Timorese consultative group that was diverse in age, gender, and education (Silove *et al.*, 2002). Piloting in preparation for the present study identified 11 items from the longer list that were specifically relevant to women of childbearing age. Each item scored on a 4-point Likert scale (1 = no problem at all, 2 = a bit of problem, 3 = moderately serious problem, 4 = a serious problem) (Silove *et al.*, 2015). Examples of items include poverty, not having enough money for food, and not having enough money for school fees. Items were assigned a score of 0 (no problem or bit of a problem) or 1 (moderately serious or a serious problem), with the addition of item scores generating a total poverty/deprivation score. We dichotomized the sample (1, 0) following an examination of the distribution of scores into those designated high (1) and low (2), with high levels of poverty being based on the cut-off of endorsing two or more items as a moderately serious or serious problem. The final index of socio-economic status, therefore, represented a composite of education (0–1), employment (0–1), and the poverty/deprivation index (1,0). The range of scores for the composite index was 0–3, a higher score indicating a greater socio-economic advantage.

### Traumatic events (TEs) for men

We assessed 23 conflict-related TEs listed in the Harvard Trauma Questionnaire (HTQ), modified to the context of Timor-Leste (Silove *et al.*, 2014). Because of the relatively young age of the sample, TEs were assessed from the onset of the humanitarian emergency (1999) through to the time of the second wave survey, which included periods of internal conflict. The timespan corresponded to when our cohort of men was in their adolescence or early adulthood, an age group that played an active role in the sequential episodes of conflict that occurred during that historical period. The most severe period of conflict after the humanitarian emergency was in 2006–07, during which factional violence led to the widespread internal displacement of populations, particularly in the Dili district, the site of our study. Items measured included traumas directed at the self and others, and of losses and separations. Typical items included political imprisonment, assault, torture, witnessing murder, exposure to atrocities, losses/separations of family or close others, and severe deprivation of medical care for self or others.

We used qualitative methods to adapt the trauma items to the local context, a process that involved iterative field testing and consultation with a Timorese committee whose membership was drawn from a range of backgrounds. During the process we established that the term for torture in the lingua franca, Tetum, encompassed abuses such as beatings and other intentional violent, retributive and punitive acts enacted by the military or their proxies (the militia), or in the civil conflict of 2006–07 by other political factions for reasons of political rivalry, retribution or revenge. In our initial bivariate analyses, we tested the torture item alone, and then a composite variable comprising the addition of all the remaining TEs (1 if endorsed, 0 if not) to assess for associations with IPV as described hereunder.

### PTSD for men

We used the most widely applied measure in the refugee and post-conflict field, the Harvard Trauma Questionnaire (HTQ), to assess symptoms of PTSD according to the fourth edition of the Diagnostic and Statistical Manual of the American Psychiatric Association (DSM-IV) (American Psychiatric Association, 1994). Each symptom is scored on a 4-point Likert scale, the summary score (1–4) representing the mean of added item scores divided by the number of items (Reynaldo, 1999). A previous convergence study in Timor-Leste showed a sound level of agreement between the HTQ PTSD symptom scale and the Structured Clinical Interview for DSM-IV (SCID) PTSD module applied independently by Australian trained psychologists: Area under the Curve (AUC) = 0.82 (95% CI: 0.71–0.94) (Liddell *et al.*, 2013). We applied a community threshold score of ⩾2.0 to allow us to dichotomize men's responses into those with and without elevated symptoms of PTSD (Silove *et al.*, 2007).

### Severe psychological distress for men

The Kessler-10 scale (K10), a measure of psychological distress, has been widely applied across cultures. The instrument consists of 10 items each scored on a 5-point Likert scale (1 = none, 2 = a little of the time, 3 = some of the time, 4 = most of the time, 5 = all the time). The measure primarily assesses depression although anxiety and somatic symptoms are included (Furukawa *et al.*, 2003). The internal reliability (Cronbach's alpha) of K10 items for men in the present sample was 0.90. A threshold of ⩾30 (the recommended international cut-off) was identified in a convergence study in Timor-Leste as providing the best balance between sensitivity and specificity when compared with a structured clinical interview (Liddell *et al.*, 2013). That threshold was used to allocate men to a high or low psychological distress group.

### Alcohol use for men

We used questions about men's alcohol use as reported by their partners. The questions are included in the World Health Organisation measure for violence against women. Questions include: (1) Does (did) your husband/partner drink (alcohol)? (2) How often does (did) he drink: very often, only sometimes, or never? In this study, we applied the cut-off of men ‘drinking very often’ as rated by their women partners to indicate likely misuse.

### Mental disturbance measured amongst men

Initial analysis showed that severe psychological distress, high PTSD symptoms (⩾2.0 on the HTQ) and misuse of alcohol were inter-correlated, representing an anticipated high level of comorbidity amongst these common symptoms and responses to trauma. For that reason, and to avoid the statistical problem of multicollinearity, we generated a composite measure of ‘mental disturbance’ in which men participants were positively identified if they met criteria for one or more of three symptom domains: threshold PTSD symptoms (⩾2.0 on the HTQ); threshold severe psychological distress (K10 ⩾30); and drinking very often as rated by women partners. For men meeting any one (or more) of these three criteria, a score of 1 was assigned for mental disturbance.

### IPV assessed amongst women

We assessed IPV according to the World Health Organization Violence Against Women Interview, which records physical, psychological, and sexual violence committed by an intimate partner. The measure has been applied in the Multi-Country Study on Women's Health and Domestic Violence across 14 countries globally (Garcia-Moreno *et al.*, 2006). The timeframe was the 12-month prevalence of IPV enacted by the current or most recent partner. Our experience in piloting combined with advice from cultural consultants led us to exclude the sexual abuse items because the topic was highly sensitive for women in a traditional and deeply religious society. In our baseline analysis, we found that three overlapping categories (severe psychological abuse alone, physical abuse alone, and a combination of both severe psychological abuse and any physical abuse) were all strongly associated with mental distress and functional impairment in women (Rees *et al.*, 2016). We, therefore, combined these categories into a severe IPV grouping, the comparison group being a combined group of women reporting no IPV or low respect/regard only. This comparison category was not significantly associated with mental distress or functional impairment. Nevertheless, it is recognised that some items reflect pervasive socially or culturally informed beliefs relating to gendered roles and expectations which may place women at higher risk of IPV in the future (Flood & Pease, 2009). It included items such as whether the spouse spends free time with the respondent, consults her on household matters; respects her and her wishes; and trusts her with money.

### Field team

The field team of 18 Timorese workers received 2 weeks of training followed by 2 months of experience field-testing and piloting measures supervised by the Australian team. Pairs of interviewers were required to achieve 100% inter-rater reliability on the symptom measures prior to conducting interviews in the field. Interviews were conducted in participants’ homes and lasted an hour. Interviews with men and women residing in the same house were conducted individually, in private and on separate days. Both were assured of confidentiality in relation to their responses.

### Ethical considerations

The study was approved by the Human Research Ethics Committee of the University of New South Wales, the Ministry of Health of Timor-Leste, and the chiefs of each village. Signed or witnessed verbal consent was obtained from all participants and interviews were conducted under conditions of strict privacy.

### Statistical analyses

We conducted bivariate analyses to test for associations of men's age, socio-economic status, torture exposure, and mental disturbance with IPV (the latter reported by women). We included variables that showed a statistically significant bivariate association in the subsequent path analysis. The path model was constructed to test the effects of the two main predictors (torture and socio-economic status), taking into account the age of male partners. The mental disturbance was located at the second tier of the model to test (1) whether torture and socio-economic status impacted directly on that outcome; and (2) whether mental disturbance, in turn, led to IPV. In addition, direct paths were allowed from torture and socio-economic status to IPV.

Given that torture, mental disturbance, and IPV were dichotomous/categorical variables, we restricted the analysis to observed variables, applying path analysis within the framework of structural equation modelling using the weighted least squares means and variance adjusted estimation method (Marcoulides, 1996; Hox, 1998; Muthén 1998–2012).

We evaluated model fit according to the conventional suite of indicators including a non-significant chi-square test; Comparative Fit Index (CFI) >0.90; the Tucker-Lewis Index (TLI) >0.90; the root mean square error of approximation (RMSEA) <0.08; and the weighted root-mean-square residual (WRMR)<0.90 (Kline, 1998; Muthén 1998–2012; Hu and Bentler 1999; Hair *et al*. 2010). Descriptive analyses were performed in SPSS version 24 (IBMCorp, Released 2016) and MPLUS 7.1 was used to test the path model (Muthén LK 1998–2012; IBMCorp, Released 2016).

## Results

### Socio-demographic and mental health characteristics

Table 1 presents descriptive data for both women and men (*n* = 870 couples). The mean age of women was 28.5 years. About a fifth (17%) of women either never attended school or had completed primary school only, nearly two thirds (58%) had completed junior/senior high school, and a quarter (25%) had obtained a university degree or other post-school qualification. Half (52%) were unemployed. About 38.5% (*n* = 335) reported physical and severe psychological abuse, a further 22.3% (*n* = 194) experienced severe psychological abuse alone (Table 1). In total, 529 (60.8%) of women fell into the severe IPV category (the index used in the path analysis), the remainder (341, 39.2%) into the low/no IPV category.

**Table 1.** Socio-demographic and mental health measures for couples (*n* = 870)

Socio-demographic characteristics and mental health indices	Couples: women and male partners (*n*=870)			
	Women		Men partners	
	Number	Col %	Number	Col %
Age				
<25 years	192	22.1	57	6.6
25–29	337	38.7	209	24.0
≥30	341	39.2	604	69.4
*Mean age (standard deviation)*		*28.5 (5.2)*		*33.6 (7.0)*
Educational status				
None or Primary School	147	16.9	151	17.4
Junior/Senior High School	502	57.7	448	51.5
Technical College/Diploma	50	5.7	101	11.6
University	171	19.7	170	19.5
Employment status				
Unemployed	459	52.8	577	66.3
Paid employment/small trade/farming	411	47.2	293	33.7
Intimate partner violence (IPV)^a^				
No IPV or low regard only	341	39.2		
Severe psychological or any physical abuse	529	60.8		NA
Mental health indices				
Post-traumatic stress disorder (PTSD): threshold ≥2.0	23	2.6	24	2.8
Kessler-10 (K10) psychological distress: threshold ≥30.0	62	7.1	45	5.2
Frequency of drinking alcohol: very often (reported by female partner)	NA		46	5.3
Any mental disturbance (satisfied threshold for PTSD or K10 or reported as drinking very often by female partner)	NA		103	11.8

a IPV items are grouped as follows. Low respect/regard only: participants included if endorsed one or more items from this group but not from forms of IPV categorized higher in the hierarchy. Example of items include spends his free time with you; consults on different household matters with you; respects you and your wishes; does not trust you with any money. Severe psychological (threatening, intimidating, and controlling): participants included if endorsed one or more items from this group. Participants may have endorsed items with low respect/regard but were not included if endorsed any physical abuse items. Items included in this group are: he is affectionate with you; jealous or angry if you talk to other men; frequently accuses you of being unfaithful; does not permit you to meet your girl friends; tries to limit your contact with your family; insists on knowing where you are at all time; humiliates you in front of others; threatens you/someone close to you with harm. Physical violence: participants were included if they endorsed one or more of: pushes you, shakes you or throws something at you; slaps you or twists your arm; punches you with something that could hurt you; kicks/drags you; tries to strangle/burn you; threatens you with a knife, gun or other type of weapon; attacks you with a knife, gun or other type of weapon; other ways your husband hurts you. Participants were included in the physical violence only group if they did not endorse any items from the threatening/jealous/controlling group. They may have endorsed items from the low respect/ regard category. Physical violence plus severe psychological (threatening, intimidating and controlling): participants were included in this group if they endorsed a minimum of one item from physical violence and one from threatening/ jealous/controlling. In addition, they may have endorsed items from lack of sharing/respect.

NA, not applicable.

The mean age of men was 33.6 years (Table 1). Half (51%) had completed junior/senior high school and a third (31%) had obtained a university degree or other post-school qualification. A third of the men (33.7%) were engaged in paid employment or farming. Over one in 11 (8.7%) reported being tortured. One out of 20 (5.2%) men reached the threshold for severe psychological distress on the K10 and 2.8% met the threshold for PTSD. About 5.3% of the women rated their male partners as drinking very often. In total, 11.8% (*n* = 103) of men met the criteria for the composite measure of mental disturbance, that is, either reaching the threshold for severe psychological distress (K10 score ⩾30.0), or PTSD (score ⩾2.0) or rated as consuming alcohol very often (Table 1).

### Bivariate analyses

In the bivariate analysis, all potential predictors were significantly associated with IPV exposure. Specifically, amongst men, younger age, lower socio-economic status, exposure to torture, and mental disturbance were statistically associated with IPV status and therefore were included in the path model (Table 2). Importantly, whereas torture was positively and statistically associated with IPV, no such relationship was evident for the composite index of other traumas. We, therefore, did not include the composite index of other traumas in the path analysis.

**Table 2.** Bivariate association of women's IPV status with their male partners’ socio-demographic characteristics and index of mental health disturbance

Socio-demographic characteristics and mental health indices of male partners	Women's IPV category (*n* = 870)			
	Combined no IPV and low respect/regard alone (*n* = 341)		Severe psychological or any physical abuse (*n* = 529)	
	Number	Col %	Number	Col %
Male Age group (in years)**				
<25 years	21	6.2	36	6.8
25–29	58	17.0	151	28.5
≥30	262	76.8	342	64.7
Mean age (years)**	*34.8*		*32.8*	
Torture**				
No	322	94.4	472	89.1
Yes	19	5.6	57	10.8
Socio-economic index (male)*				
0 (lowest socio-economic status)	15	4.4	36	6.8
1	120	35.2	213	40.3
2	127	37.2	198	37.4
3 (highest socio-economic status)	79	23.2	82	15.5
Any mental disturbance (threshold for PTSD or K10 or drinking alcohol very often) for male**				
No	319	93.5	448	84.7
Yes	22	6.5	81	15.3

*Women's IPV rate significantly differ at *p*<0.05; **Significantly differ at *p*<0.01.

### Path analysis

**Fig. 1.**
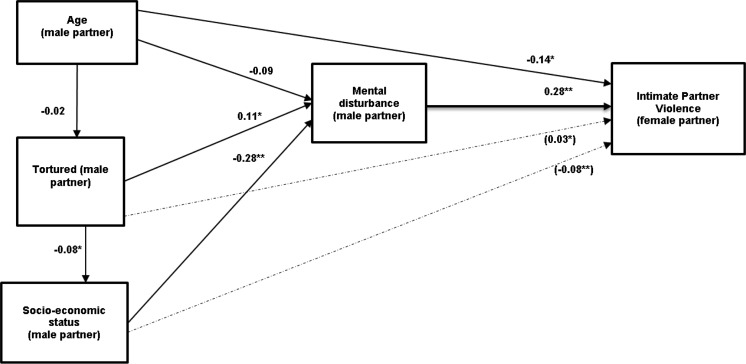
Path analysis examining effects of men's age, torture exposure, and socio-economic status leading to IPV via the composite index of mental disturbance (based on symptoms of PTSD, depression/anxiety, alcohol abuse). *Notes:* Age (male partner): male partner age (continuous score); Torture (male partner) (no=0, yes=1); Socio-economic status (male partner): based on education, employment and ongoing adversity (0–3); Mental disturbance (male partner): threshold for PTSD or K10 or rated by female partner as drinking very often (no=0, yes=1); IPV (reported by female partner): Combined no IPV or low respect/regard alone=0, Severe psychological and/or any physical abuse=1. **( .-.-.-.)** Dash lines show significant indirect paths. *Indirect Path:* torture → mental disturbance → IPV (standardized coefficient= 0.031; *p* = 0.041). socio-economic status → mental disturbance → IPV (standardized coefficient=−0.080; *p* = 0.001). *Model summary:* CFI (Comparative Fit Index): 1.00; TLI (Tucker-Lewis Index): 1.00; WRMR (Weighted Root Mean Square Residual): 0.32; RMSEA (90% CI of RMSEA): 0.004 (0.00–0.090). χ^2^ Test of Model Fit: Value 1.013; *p* = 0.314. **Standardized coefficients significant at p<0.01; *Standardized coefficients significant at *p* < 0.05. All the variables included in the analyses are based on observed data with no use of latent variables.

Figure 1 displays the path model with standardized coefficients for direct (unbroken lines) and indirect pathways (broken lines). The model yielded a very good fit to the data (CFI = 1.00, TLI = 1.00, RMSEA = 0.004, and WRMR = 0.32).

Lower socio-economic status was significantly associated with past torture in men (standardized path coefficient = −0.08; *p* < 0.05). The three key predictors of mental disturbance in men were younger age (standardized path coefficient = −0.09; *p* < 0.05); torture exposure (standardized path coefficient = 0.11; *p* < 0.05) and lower socio-economic status (standardized path coefficient = −0.28; *p* < 0.01). Mental disturbance in turn predicted IPV (standardized path coefficient = 0.28; *p* < 0.01). Younger age of men also predicted IPV (standardized path coefficient = −0.14; *p* < 0.05). In addition, indirect pathways via mental disturbance to IPV were evident for torture (standardized indirect path coefficient = 0.03; *p* < 0.05) and lower socio-economic status (standardized indirect path coefficient = −0.08; *p* < 0.01) (Fig. 1).

## Discussion

Our findings support the hypothesis that male survivors of torture are more likely to perpetrate IPV and that the presence of common comorbid mental health symptoms (PTSD, depression and anxiety, and substance misuse) play a key role in mediating that effect. The findings, therefore, suggest that of all the traumas of mass conflict, torture may have a specific role in creating a risk of IPV in families in the post-conflict period. As predicted, torture and lower socio-economic status were related, supporting the hypothesis that survivors of the former abuse are more likely to experience poverty in the post-conflict period. In addition, lower socio-economic status predicted mental disturbance which in turn led to IPV.

Our findings offer further support for a cycle of violence model within vulnerable families in post-conflict societies (Rees *et al.*, 2015). The core component proposed in the model is that exposure to gross human rights violations, in this case, torture, increases risk of future violence within the family. According to our path model, this effect is substantially mediated by the presence of common comorbid symptoms of mental disturbance, including PTSD, anxiety and depression, and alcohol misuse amongst male survivors of torture. The flow-on psychosocial effects for families and society, in general, are therefore of grave concern given established knowledge that IPV is itself a major source of mental disturbance and psychosocial dysfunction in survivor women, including direct evidence from Timor-Leste (Rees *et al.*, 2016). The cycle of violence model supported by data reported here assists in creating an important bridge between research into the individual mental health impacts of torture, and the potential society-wide and familial effects that such abuses can perpetuate in the post-conflict environment (Widom & Maxfield, 2001; Catani, 2010; Rees *et al.*, 2015).

The prevalence of IPV in our study was high, with this form of abuse being reported by two-thirds of participating women. Clearly, the figures indicate that torture can only be one of several factors contributing to the pervasive problem of IPV in post-conflict Timor-Leste and most likely, in other similarly affected countries worldwide. It seems probable that society-wide factors such as a culture of patriarchy and gender inequality are major contributors to this problem (Wallerstein, 1992; Heise *et al.*, 1994; Jewkes, 2002; Dunkle *et al.*, 2004; Hunnicutt, 2009; Pease, 2012; Gupta *et al.*, 2013; Fleming *et al.*, 2015), typical of other traditional societies where rates of IPV have been found to be similarly high (Heise, 1998; Jewkes, 2002; Dunkle *et al.*, 2004; Yodanis, 2004; Fulu *et al.*, 2013; Vichealth, 2014).

The finding that younger age in men is associated with mental disturbance and the pathway to IPV can be explained by contemporary stressors on younger men in a country recovering from conflict, with few opportunities for employment or other forms of social advancement. Our finding, therefore, suggests the need for a greater focus on education and awareness related to IPV laws and social expectations amongst younger men.

In speculating about the precise mechanisms whereby torture leads to the enactment of IPV, we caution against the risk of stigmatizing torture survivors, particularly given that they are vulnerable to social exclusion and marginalization in society at large. An ecological perspective underpinning the cycle of violence model emphasizes the responsibility of the system of persecution in initiating the cycle of violence, implicating all actors including the ruling regime, the military apparatus, and its proxies (in Timor-Leste, the militia) that together orchestrated, oversaw, and directed torture as a means of repression. In that context, the torture survivor, particularly in cases where there was no prior history of violence against women, is the first of a sequence of victims. At the same time, no perpetrator of IPV can ever be excused, and all sanctions and responses to protect women and children need to be applied equally, whatever the background factors that lead to the abuse. At a community-wide level, in the case of torture survivors, the focus should be on violence prevention by way of early identification and support for men at risk; provision of timely work, educational, and financial assistance; and programs aimed at harmonizing the re-entry of the male survivor back into the family and broader community (Pease & Flood, 2008; Vichealth, 2014). The adage that violence begets violence is no more apt than in this situation, with the disruption of the cycle of violence requiring a sensitive and comprehensive approach (Brendtro & Long, 1994; Widom & Maxfield, 2001).

At a therapeutic level, our findings serve as an instructive reminder to the trauma field of the importance of identifying and addressing the mental health impacts of torture both on the survivor and the wider family. In addition to providing specific interventions for PTSD and alcohol misuse, torture survivors often require a broader psychotherapeutic approach which focuses on problems of self-image, threatened masculinity, the expression and management of aggression, and altered roles in the family and society. A vital component of any intervention is to include psycho-education regarding gender equality and respect, particularly within the family, issues that may be reinforced in separate venues, for example, in men's groups (Pease, 2012). Importantly, at a society-wide level, recognition of the contribution and sacrifices made by militants (in the case of Timor-Leste, those engaged in the struggle for national independence), can play an important restitutive role.

Women known to be living with torture survivors should receive special attention in IPV screening programs. Included in the assessment should be an evaluation of the financial status of the family, given that our study supports existing evidence demonstrating an association between financial hardship and IPV (Heise *et al.*, 1994; Jewkes, 2002). The combination of financial hardship and the male partner being a torture survivor should alert services to the multi-pronged needs that these families confront. Promoting the economic capacity of women in these situations by providing them with resources and skills to enter the workplace has the added advantage of protecting against IPV (Flood & Pease, 2009). Nevertheless, the engagement of families to provide such support for women will require great sensitivity, and involve a multidimensional approach that focuses on the personal and interpersonal factors associated with IPV and torture, while attending to issues of safety and the need for genuine empowerment of both partners (Gupta *et al.*, 2013). In that respect, there is a pressing need to design and test culturally and contextually relevant couple- and family-focused programs that address the sequential traumas of past torture, ongoing IPV, and related mental distress in post-conflict settings such as Timor-Leste (Fisher *et al.*, 2016; Silove *et al.*, 2017).

Our study had several strengths and limitations. This is the first systematic study in the field focusing on the association between torture and IPV, in this instance undertaken amongst a large community cohort of couples in a low-income, post-conflict country. A major strength of the study is that the data were collected confidentially from men and women partners in separate interviews on different occasions. Specifically, men responded to questions regarding their socio-economic status, past torture, and mental health independently of their female partner's accounts of recent or ongoing IPV and observed alcohol use in the male. There was a lower response rate amongst men, typical of epidemiological studies where women are the primary participants. Torture survivors may have been less likely to participate because of their suspicion of authority figures. That factor, and the risk of under-reporting both torture and IPV, may have led to an underestimation of the strength of the relationship between torture and IPV. We did not establish whether men were perpetrators of IPV prior to being tortured, although the likelihood of that being the case for many participants was low given that most were youth or young adults at the time of the major periods of conflict when these abuses occurred. Another limitation is the omission of sexual violence questions. Sexual violence in intimate relationships is common and often co-occurs with physical abuse and psychological abuse. The non-inclusion of sexual violence items could only serve to reduce the prevalence estimation. Our data are also limited to one district of Timor-Leste, requiring replication in other settings in the country and internationally. The cross-sectional nature of the study cautions against drawing causal inferences regarding the theoretical pathways tested in the model. We note, however, that longitudinal methodologies in this field are virtually impossible to implement given the difficulty of predicting future episodes of conflict and persecution in any given country. The torture definition applied in the study was limited to one item in the Harvard Trauma Questionnaire, indicating the need to replicate the study with a more comprehensive assessment. Nevertheless, the item was carefully evaluated for its contextual relevance in earlier qualitative research undertaken by the team. In general, we acknowledge that there may be transcultural errors in the adaptation and translation of our measures, despite our systematic application of qualitative and quantitative methods to achieve contextual congruence in the Timor-Leste context. We finally note that the study was conducted amongst partners with young children and the path to IPV may not be generalizable in cases where there are no children, or where there are older children.

In conclusion, our data provide the first systematic evidence of an association between torture and IPV in a low-income, post-conflict country. The model also confirmed that the younger age of men and socio-economic deprivation played a considerable role in the paths leading to domestic abuse. Importantly, a composite index of mental disturbance including symptoms of PTSD, anxiety, and depression, and alcohol abuse, substantially accounted for the association of torture with IPV. Our findings, therefore, provide crucial empirical support for the role of torture as a factor in the emerging cycle of violence model which aims to explain some of the psychosocial problems encountered by women and families in the wider society and in post-conflict settings worldwide. The high prevalence of IPV identified here and in other low-income, post-conflict countries, also underscores the importance of addressing broader social and structural factors that explicitly or implicitly sanction men's use of violence against women in these societies.
