# Risk of perpetrating intimate partner violence amongst men exposed to torture in conflict-affected Timor-Leste – CORRIGENDUM

**DOI:** 10.1017/gmh.2018.23

**Published:** 2018-08-03

**Authors:** Susan Rees, Mohammed Mohsin, Alvin Kuowei Tay, Zachary Steel, Natalino Tam, Zelia da Costa, Cesarina Soares, Wietse Tol, Valsamma Eapen, Mark Dadds, Derrick Silove

Keywords: trauma; intimate partner violence; mental disturbance; torture; corrigendum

The references Pease & Flood, 2008 and Vichealth, 2014 were mistakenly added following the paragraph:

At a community-wide level, in the case of torture survivors, the focus should be on violence prevention by way of early identification and support for men at risk; provision of timely work, educational, and financial assistance; and programs aimed at harmonizing the re-entry of the male survivor back into the family and broader community.
